# Supplementary material for: Differential Tractography: A Biomarker for Neuronal Function in Neurodegenerative Disease
Source: medRxiv. 2024 Aug 26:2024.08.25.24312255. Preprint. [Version 1] doi: 10.1101/2024.08.25.24312255 (PMC11451749; doi:10.1101/2024.08.25.24312255)
Supplement: Supplement 1 [file NIHPP2024.08.25.24312255v1-supplement-1.pdf]

medRxiv preprint doi: https://doi.org/10.1101/2024.08.25.24312255; this version posted August 26, 2024. The copyright holder for this preprint (which was not certified by peer review) is the author/funder, who has granted medRxiv a license to display the preprint in perpetuity. This article is a US Government work. It is not subject to copyright under 17 USC 105 and is also made available for use under a CC0 license.

Differential Tractography: A Biomarker for Neuronal Function in Neurodegenerative Disease
Supplementary Material

Table of Contents

Methods……………………………………………………………………………………..…*18*

Supplement A: Natural History Study Age-Matched Methodology……………..………………*18*

Supplement B: Diffusion Weighted Imaging (DWI) Sequence Parameters and Processing……*21*

Results………………………………………………………………………………………...*23*

Supplement C: Fractional Anisotropy Thresholds Correlate with CGI-C..……………………..*23*

References..……………………………………………………………………………………...*27*

medRxiv preprint doi: https://doi.org/10.1101/2024.08.25.24312255; this version posted August 26, 2024. The copyright holder for this preprint (which was not certified by peer review) is the author/funder, who has granted medRxiv a license to display the preprint in perpetuity.
This article is a US Government work. It is not subject to copyright under 17 USC 105 and is also made available for use under a CC0 license.

Supplementary Methods

Supplement A: Natural History Study Age-Matched Methodology

NHGRI Natural History Study (NCT00029965)[1]

Study description
This is a natural history study that will evaluate any patient with enzyme- or DNA-confirmed GM1 or GM2 gangliosidosis, sialidosis or galactosialidosis. Patients may be evaluated every 6 months for infantile onset disease, yearly for juvenile onset and approximately every two years for adult-onset disease as long as they are clinically stable to travel. Data will be evaluated serially for each patient and cross-sectionally for patients of similar ages and genotypes. Genotype-phenotype correlations will be made where possible although these are rare disorders and the majority of the patients are compound heterozygotes.

Objectives

- To study the natural history and progression of neurodegeneration in individuals with glycosphingolipid storage disorders (GSL), GM1 and GM2 gangliosidosis, and glycoprotein (GP) disorders including sialidosis and galactosialidosis using clinical evaluation of patients and patient/parent surveys.
- To develop sensitive tools for monitoring disease progression.
- To identify biological markers in blood, cerebrospinal fluid, and urine that correlate with disease severity and progression and can be used as outcome measures for future clinical trials.
- To further understand and characterize the mechanisms of neurodegeneration in GSL and GP storage disorders across the spectrum of disease beginning with ganglioside storage in fetal life.

Study Population
Patients with enzyme- or DNA-confirmed GM1 or GM2 gangliosidosis, sialidosis or galactosialidosis. Accrual ceiling is 200 participants, with no exclusions based on age, gender, demographic group, or demographic location. Patients included in our study are those who were seen at the NIH Clinical Center or who only sent in blood samples or who complete the questionnaire or provided head circumference measures.

Inclusion Criteria
- Individuals greater than 6 months of age with GM1 or GM2 gangliosidosis documented by enzyme deficiency and/or mutation analysis in a CLIA-approved laboratory

Exclusion Criteria

- Individuals who in the opinion of the principal investigator are too medically fragile to travel safely to the NIH for evaluation
- Individuals unable to comply with the protocol

medRxiv preprint doi: https://doi.org/10.1101/2024.08.25.24312255; this version posted August 26, 2024. The copyright holder for this preprint (which was not certified by peer review) is the author/funder, who has granted medRxiv a license to display the preprint in perpetuity. This article is a US Government work. It is not subject to copyright under 17 USC 105 and is also made available for use under a CC0 license.

NHGRI Natural History Study Age Matched Cohort

The data included in this investigation represents a subset of the natural history study patients. This study includes only patients who had a confirmed GM1 Gangliosidosis diagnosis, excluding those with other glycosphingolipid storage disorders, glycoprotein disorders, and GM2 Gangliosidosis who were a part of the larger natural history cohort (**Table A1**). Patients were selected for the longitudinal analysis cohort based on having multiple diffusion weighted imaging scans with corresponding cognitive global impression (CGI) scores (**Table A2**). Patients were selected for the age-matched cohort based on their baseline scan age and follow-up scan (table). Only patients who had repeated diffusion weighted imaging scans within the range of the normal controls (2.5 years old – 16 years old) were included (**Table A3**).

Table A1. Natural History Study Age Matched Cohort (n = 10), specific ages redacted per MedArXiv requirements

Participant	Baseline Age (years old)	Oldest Follow-up (years old)	DWI Interval (years)	GM1 Subtype
NHS 10	11-15	11-15	2.2	Juvenile
NHS 20	11-15	11-15	3.5	Juvenile
NHS 54	0-5	0-5	1	Juvenile
NHS 58	6-10	11-15	4	Juvenile
NHS 69	0-5	6-10	1.1	Juvenile
NHS 72	6-10	6-10	0.95	Late-Infantile
NHS 73	6-10	6-10	1.2	Late-infantile
NHS 84	0-5	6-10	1.9	Late-infantile
NHS 93	6-10	6-10	1	Juvenile
NHS 94	6-10	6-10	1	Juvenile
Mean ± SD	8.45 ± 3.20	10.24 ± 3.91	1.79 ± 1.12	N/A

Table A2. Natural History Study Longitudinal Analysis Cohort (n = 16), specific ages redacted per MedArXiv requirements

Participant	GM1 Sub-type	Baseline Age (years old)	Scan #2 Age (years old)	Scan #3 Age (years old)	Average DWI Interval (years)	Number of DWI Scans
NHS 03	Juv	21-25	21-25	N/A	1.0	2
NHS 09	Juv	11-15	16-20	21-25	3.1	3
NHS 10	Juv	11-15	11-15	N/A	2.2	2
NHS 11	Juv	11-15	16-20	16-20	2.05	3
NHS 20	Juv	11-15	11-15	11-15	1.75	3
NHS 25	Juv	11-15	16-20	16-20	2.25	3
NHS 26	Juv	11-15	16-20	N/A	4.6	2
NHS 28	Juv	16-20	21-25	N/A	2.3	2
NHS 54	Juv	0-5	0-5	N/A	1.0	2
NHS 58	Juv	5-10	11-15	11-15	2.0	3
NHS 69	Juv	0-5	6-10	N/A	1.1	2

medRxiv preprint doi: https://doi.org/10.1101/2024.08.25.24312255; this version posted August 26, 2024. The copyright holder for this preprint (which was not certified by peer review) is the author/funder, who has granted medRxiv a license to display the preprint in perpetuity. This article is a US Government work. It is not subject to copyright under 17 USC 105 and is also made available for use under a CC0 license.

NHS 72	LI	6-10	6-10	N/A	0.95	2
NHS 73	LI	6-10	6-10	N/A	1.2	2
NHS 84	LI	0-5	6-10	N/A	1.9	2
NHS 93	Juv	6-10	6-10	N/A	1.0	2
NHS 94	Juv	6-10	6-10	N/A	1.0	2

Table A3. Normal Control Age Matched Cohort (n = 32), specific ages redacted per MedArXiv requirements

Participant	Baseline Age (years old)	Oldest Follow-up (years old)	DWI Interval (years)	Database
10073	0-5	0-5	1.3528	Calgary
10007	0-5	0-5	1.3528	Calgary
10066	0-5	0-5	1.39	Calgary
10054	0-5	6-10	2.4163	Calgary
10148	0-5	6-10	2.1889	Calgary
10109	0-5	6-10	2.1306	Calgary
10022	0-5	6-10	2.37334	Calgary
10025	0-5	6-10	2.05	Calgary
10027	0-5	6-10	1.8889	Calgary
10090	0-5	6-10	2.1278	Calgary
10020	6-10	6-10	1.075	Calgary
10087	6-10	6-10	0.8889	Calgary
10161	6-10	6-10	1.0166	Calgary
360	6-10	11-15	2	QTAB
410	11-15	11-15	2	QTAB
411	6-10	11-15	2	QTAB
376	6-10	6-10	1	QTAB
378	6-10	11-15	2	QTAB
405	6-10	6-10	2	QTAB
200	11-15	11-15	2	QTAB
197	11-15	11-15	2	QTAB
190	11-15	11-15	1	QTAB
186	11-15	11-15	3	QTAB
183	11-15	11-15	3	QTAB
172	11-15	11-15	2	QTAB
173	11-15	11-15	2	QTAB
162	11-15	11-15	2	QTAB
158	11-15	11-15	1	QTAB
152	11-15	11-15	2	QTAB
157	11-15	11-15	2	QTAB
156	11-15	11-15	2	QTAB
155	11-15	11-15	2	QTAB
Mean ± SD	8.54 ± 3.16	10.39 ± 3.32	1.85 ± 0.54	N/A

medRxiv preprint doi: https://doi.org/10.1101/2024.08.25.24312255; this version posted August 26, 2024. The copyright holder for this preprint (which was not certified by peer review) is the author/funder, who has granted medRxiv a license to display the preprint in perpetuity. This article is a US Government work. It is not subject to copyright under 17 USC 105 and is also made available for use under a CC0 license.

Supplement B: Diffusion Weighted Imaging (DWI) Sequence Parameters and Processing

Natural History Study (NHS) Patients[1]
A Philips Achieva 3T system equipped with an 8-channel SENSE head coil was used to scan all Natural History Study patients. DTI images were acquired with the following parameters for NHS: TR/TE=6400/100 ms, 32-gradient directions, b-values=0 and 1000 s/mm^2, slice thickness=2.5 mm, acquisition matrix=128×128, NEX=1, FOV=24 cm.

Calgary Normal Controls (NC)[2]
A General Electric 3T MR750w system and a 32-channel head coil was used for scanning all Calgary normal controls using a single shot spin echo-planar imaging sequence. DTI images were acquired with the following parameters for Calgary normal controls: TR/TE=6750/79 ms, FOV=20 cm, 30 gradient encoding directions at b=0 and 750 s/mm^2.

Queensland Normal Controls(NC)[3]
A 3T Magnetom Prisma (Siemens Medical Solutions, Erlangen) and a 64-channel head coil at the Centre for Advanced Imaging, University of Queensland using a multi-shell with an anterior-posterior phase encoding direction. DTI images were acquired with the following parameters for Queensland normal controls: TR/TE= 3800/70 ms, voxel size=2mm x 2mm x 2mm,23-gradient directions, b-values=0, 1,000, and 3,000 s/mm^2, slice thickness=2 mm, FOV=244x244mm.

DWI Preprocessing ***(Fig. B1***)
DWI was first converted from DICOM to a NIFTI file using *dcm2niix* where the b-values and b-vectors files were acquired[4]. DWI at all timestamps was preprocessed for artifacts, eddy currents, motion, and susceptibility induced distortions using MRtrix3's (MRtrix, v3.0.4)[5] *dwifslpreproc*[6-8] command utilizing the *dwi2mask*[9] function followed by FSL's (FSL, v6.0.5) *eddy*[7] and *topup*[7,8] functions. Preprocessed data was imported into DSI Studio (DSI Studio, v2023) where imaging was quality checked for bad slices, a U-Net mask was created, and generalized q-sampling imaging (GQI) reconstruction was performed with a diffusion sampling length ratio of 1.25[10].

DWI Processing ***(Fig. B2)***
First, the fractional anisotropy (FA) map of the baseline image was exported as a NIFTI file. Whole brain fiber tractography was then performed on the baseline image with 1,000,000 seeds, a step size of 1 mm, an angular threshold of 60, minimum tract size of 20 mm, and a maximum tract size of 200 mm. Differential tractography was then performed on each subsequent follow-up scan in comparison with the baseline image where fiber tract gains and losses were calculated using 10%, 20%, 30%, 40%, and 50% fractional anisotropy thresholds. Fiber tract gains were determined where the difference in FA between the follow-up and the baseline image exceeded the threshold $\frac{Scan_1 - Scan_2}{Scan_1}$. Fiber tract losses were determined where the difference in FA between the baseline and the follow-up image exceeded the threshold utilizing the equation $\frac{Scan_2 - Scan_1}{Scan_2}$. Differential tractography was calculated with the following parameters: angular threshold=60, step size=1 mm, tracts < 20 mm or > 200 mm were discarded, and 1,000,000 seeds were placed.

medRxiv preprint doi: https://doi.org/10.1101/2024.08.25.24312255; this version posted August 26, 2024. The copyright holder for this preprint (which was not certified by peer review) is the author/funder, who has granted medRxiv a license to display the preprint in perpetuity. This article is a US Government work. It is not subject to copyright under 17 USC 105 and is also made available for use under a CC0 license.

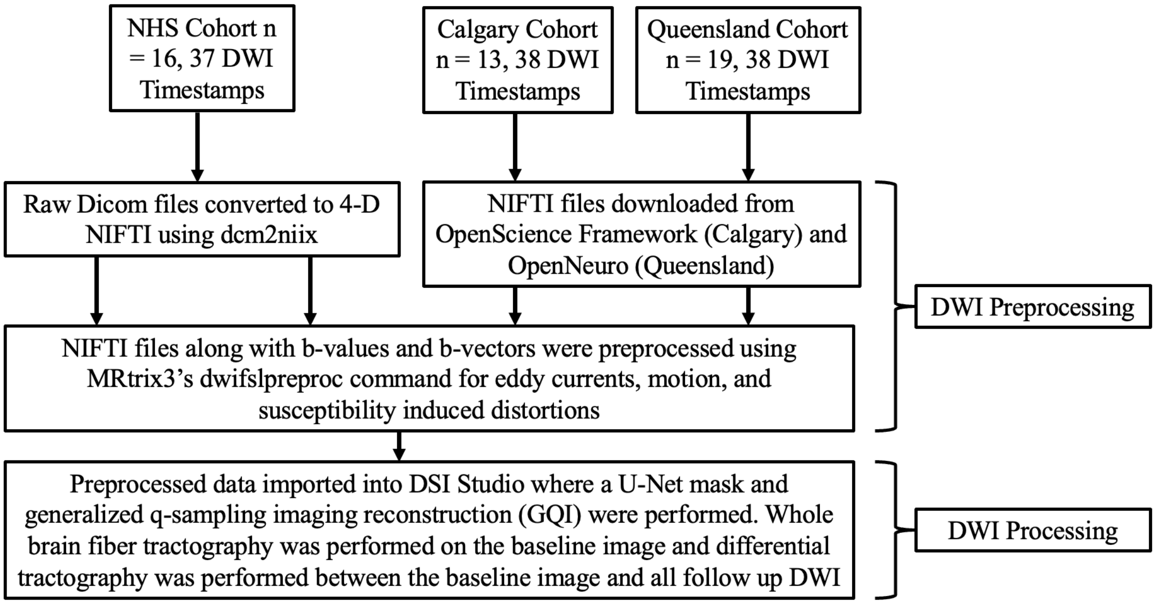

Figure B1. DWI preprocessing pipeline.

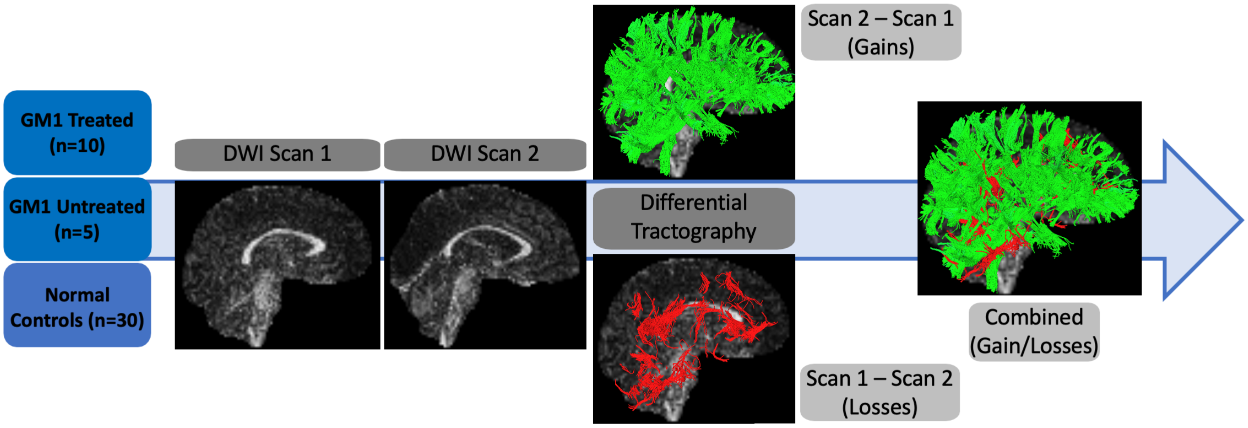

Figure B2. Differential Tractography overview.

medRxiv preprint doi: https://doi.org/10.1101/2024.08.25.24312255; this version posted August 26, 2024. The copyright holder for this preprint (which was not certified by peer review) is the author/funder, who has granted medRxiv a license to display the preprint in perpetuity. This article is a US Government work. It is not subject to copyright under 17 USC 105 and is also made available for use under a CC0 license.

Supplementary Results

Supplement C: FA Thresholds on CGI-C

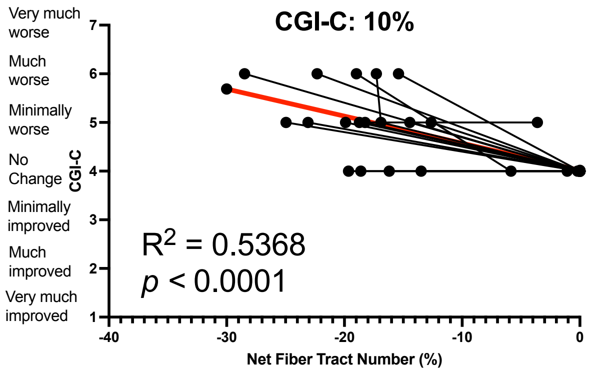

Figure C1. Differential Tractography correlations of net fiber tract number with CGI-C change scores with GM1 patients at a 10% fractional anisotropy threshold.

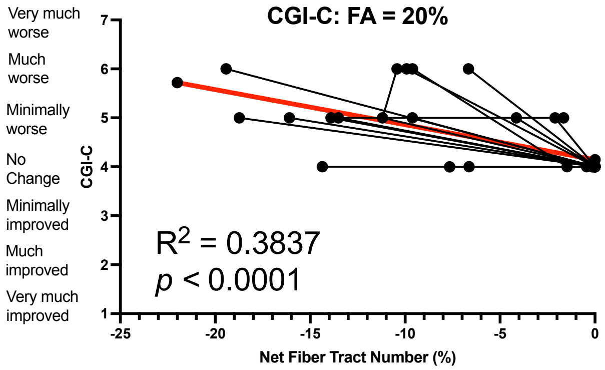

Figure C2. Differential Tractography correlations of net fiber tract number with CGI-C change scores with GM1 patients at a 20% fractional anisotropy threshold.

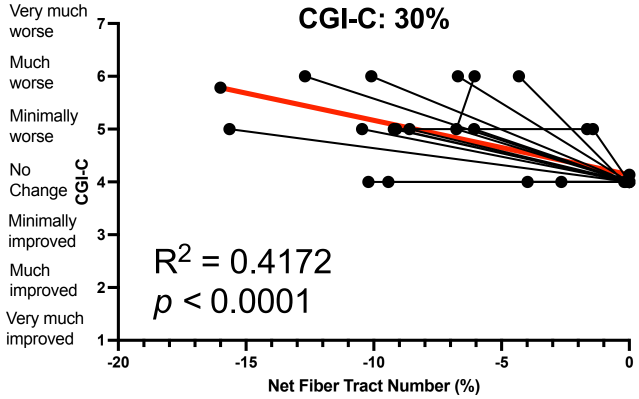

Figure C3. Differential Tractography correlations of net fiber tract number with CGI-C change scores with GM1 patients at a 30% fractional anisotropy threshold.

medRxiv preprint doi: https://doi.org/10.1101/2024.08.25.24312255; this version posted August 26, 2024. The copyright holder for this preprint (which was not certified by peer review) is the author/funder, who has granted medRxiv a license to display the preprint in perpetuity.
This article is a US Government work. It is not subject to copyright under 17 USC 105 and is also made available for use under a CC0 license.

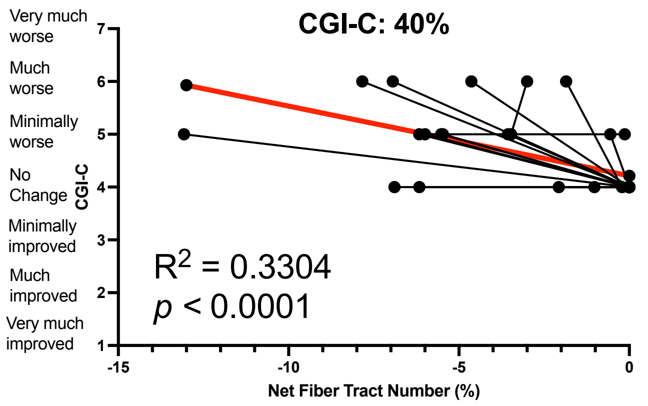

Figure C4. Differential Tractography correlations of net fiber tract number with CGI-C change scores with GM1 patients at a 40% fractional anisotropy threshold.

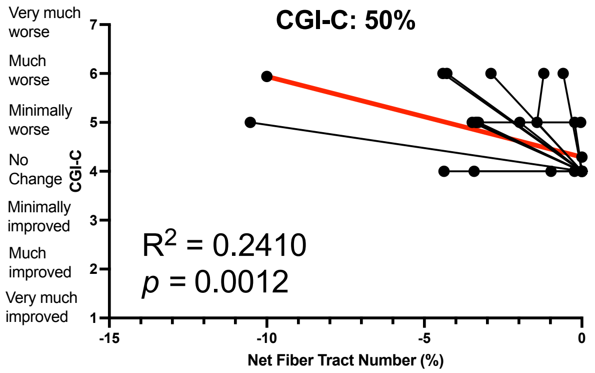

Figure C5. Differential Tractography correlations of net fiber tract number with CGI-C change scores with GM1 patients at a 50% fractional anisotropy threshold.

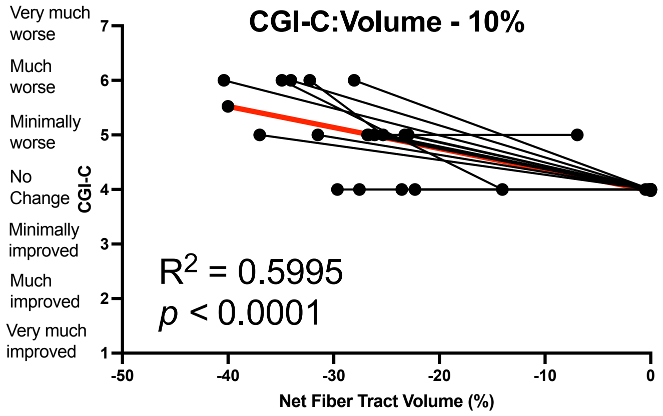

Figure C6. Differential Tractography correlations of net fiber tract volume with CGI-C change scores with GM1 patients at a 10% fractional anisotropy threshold.

medRxiv preprint doi: https://doi.org/10.1101/2024.08.25.24312255; this version posted August 26, 2024. The copyright holder for this preprint (which was not certified by peer review) is the author/funder, who has granted medRxiv a license to display the preprint in perpetuity.
This article is a US Government work. It is not subject to copyright under 17 USC 105 and is also made available for use under a CC0 license.

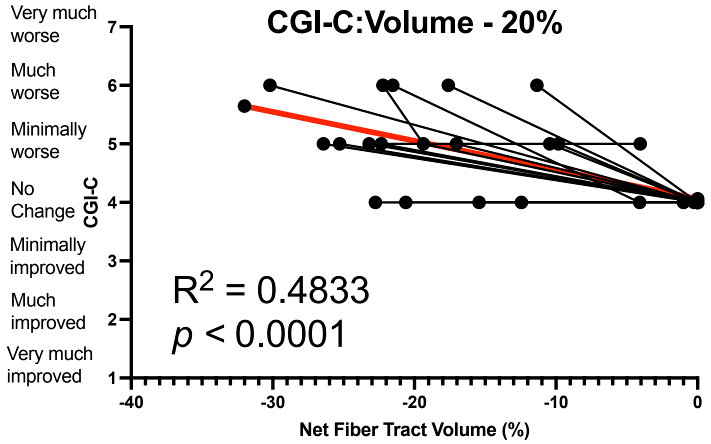

Figure C7. Differential Tractography correlations of net fiber tract volume with CGI-C change scores with GM1 patients at a 20% fractional anisotropy threshold.

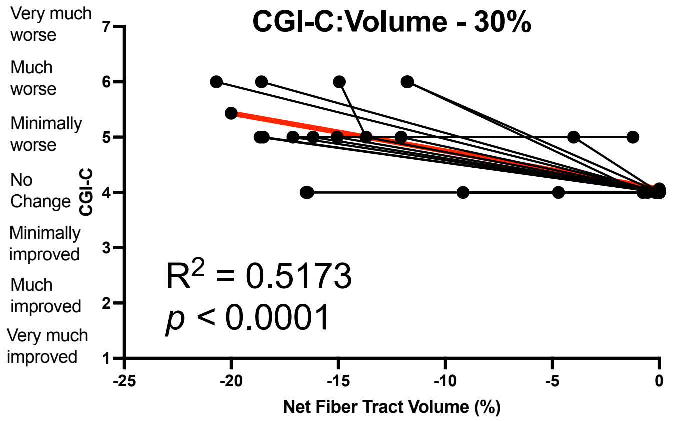

Figure C8. Differential Tractography correlations of net fiber tract volume with CGI-C change scores with GM1 patients at a 30% fractional anisotropy threshold.

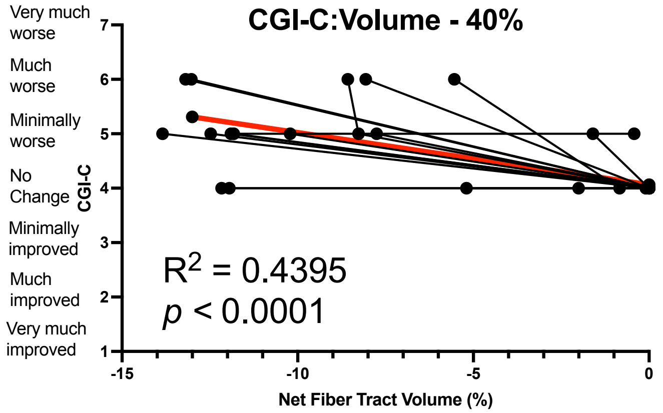

Figure C9. Differential Tractography correlations of net fiber tract volume with CGI-C change scores with GM1 patients at a 40% fractional anisotropy threshold.

medRxiv preprint doi: https://doi.org/10.1101/2024.08.25.24312255; this version posted August 26, 2024. The copyright holder for this preprint (which was not certified by peer review) is the author/funder, who has granted medRxiv a license to display the preprint in perpetuity.
This article is a US Government work. It is not subject to copyright under 17 USC 105 and is also made available for use under a CC0 license.

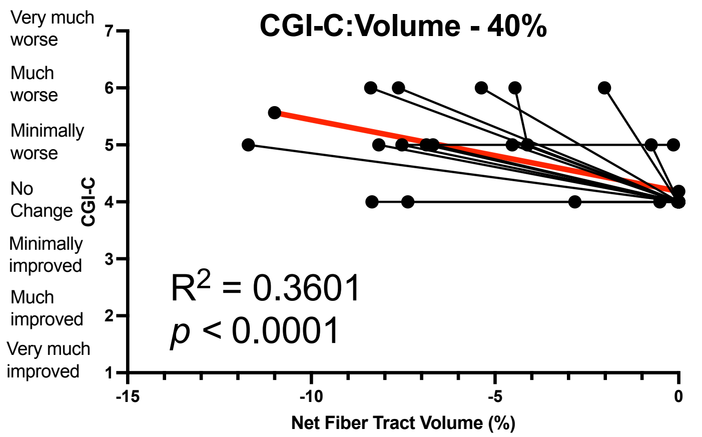

Figure C10. Differential Tractography correlations of net fiber tract volume with CGI-C change scores with GM1 patients at a 50% fractional anisotropy threshold.

Table C1. Correlations between net fiber tract number and net fiber tract volume with longitudinal CGI-C scores at varying fractional anisotropy thresholds.

Metric	FA Threshold	χ^2	R^2	*p*-value
Net Fiber Tract Number	10%	29.04	0.5368	$p < 0.0001$
Net Fiber Tract Number	20%	18.31	0.3837	$p < 0.0001$
Net Fiber Tract Number	30%	20.41	0.4172	$p < 0.0001$
Net Fiber Tract Number	40%	15.19	0.3304	$p < 0.0001$
Net Fiber Tract Number	50%	10.46	0.241	$p = 0.0012$
Net Fiber Tract Volume	10%	34.48	0.5995	$p < 0.0001$
Net Fiber Tract Volume	20%	36.58	0.4176	$p < 0.0001$
Net Fiber Tract Volume	30%	27.49	0.5173	$p < 0.0001$
Net Fiber Tract Volume	40%	21.88	0.4395	$p < 0.0001$
Net Fiber Tract Volume	50%	16.90	0.3601	$p < 0.0001$

medRxiv preprint doi: https://doi.org/10.1101/2024.08.25.24312255; this version posted August 26, 2024. The copyright holder for this preprint (which was not certified by peer review) is the author/funder, who has granted medRxiv a license to display the preprint in perpetuity.
This article is a US Government work. It is not subject to copyright under 17 USC 105 and is also made available for use under a CC0 license.

Supplementary References

1. National Human Genome Research Institute. Natural History of Glycosphingolipid Storage Disorders and Glycoprotein Disorders ClinicalTrials.gov identifier: NCT00029965. Updated August 7, 2024. Accessed August 12, 2024. https://clinicaltrials.gov/study/NCT00029965
2. Reynolds JE, Long X, Paniukov D, Bagshawe M, Lebel C. Calgary Preschool magnetic resonance imaging (MRI) dataset. Data Brief. 2020 Jan 31;29:105224. doi: 10.1016/j.dib.2020.105224. PMID: 32071993; PMCID: PMC7016255
3. Strike, L.T., Hansell, N.K., Chuang, KH. et al. The Queensland Twin Adolescent Brain Project, a longitudinal study of adolescent brain development. Sci Data 10, 195 (2023). https://doi.org/10.1038/s41597-023-02038-w
4. Li X, Morgan PS, Ashburner J, Smith J, Rorden C. The first step for neuroimaging data analysis: DICOM to NIfTI conversion. J Neurosci Methods. 2016 May 1;264:47-56. doi: 10.1016/j.jneumeth.2016.03.001. Epub 2016 Mar 2. PMID: 26945974.
5. Tournier, J. D., Smith, R., Raffelt, D., Tabbara, R., Dhollander, T., Pietsch, M., Christiaens, D., Jeurissen, B., Yeh, C. H., & Connelly, A. (2019). MRtrix3: A fast, flexible and open software framework for medical image processing and visualisation. NeuroImage, 202, 116137. https://doi.org/10.1016/j.neuroimage.2019.116137
6. Andersson, J. L. R., & Sotiropoulos, S. N. (2016). An integrated approach to correction for off-resonance effects and subject movement in diffusion MR imaging. NeuroImage, 125, 1063–1078. https://doi.org/10.1016/j.neuroimage.2015.10.019
7. Smith, S. M., Jenkinson, M., Woolrich, M. W., Beckmann, C. F., Behrens, T. E., Johansen-Berg, H., Bannister, P. R., De Luca, M., Drobnjak, I., Flitney, D. E., Niazy, R. K., Saunders, J., Vickers, J., Zhang, Y., De Stefano, N., Brady, J. M., & Matthews, P. M. (2004). Advances in functional and structural MR image analysis and implementation as FSL. NeuroImage, 23 Suppl 1, S208–S219. https://doi.org/10.1016/j.neuroimage.2004.07.051
8. Andersson, J. L., Skare, S., & Ashburner, J. (2003). How to correct susceptibility distortions in spin-echo echo-planar images: application to diffusion tensor imaging. NeuroImage, 20(2), 870–888. https://doi.org/10.1016/S1053-8119(03)00336-7
9. Cox R. W. (1996). AFNI: software for analysis and visualization of functional magnetic resonance neuroimages. Computers and biomedical research, an international journal, 29(3), 162–173. https://doi.org/10.1006/cbmr.1996.0014
10. Yeh, F. C., Wedeen, V. J., & Tseng, W. Y. (2010). Generalized q-sampling imaging. IEEE transactions on medical imaging, 29(9), 1626–1635. https://doi.org/10.1109/TMI.2010.2045126.
